# The Effect of Augmented Reality and Virtual Reality on Inducing Anxiety for Exposure Therapy: A Comparison Using Heart Rate Variability

**DOI:** 10.1155/2018/6357351

**Published:** 2018-11-25

**Authors:** Chai-Fen Tsai, Shih-Ching Yeh, Yanyan Huang, Zhengyu Wu, Jianjun Cui, Lirong Zheng

**Affiliations:** ^1^Taipei Veterans General Hospital, Taipei 11217, Taiwan; ^2^Fudan University, Shanghai 200433, China; ^3^Huashan Hospital, Fudan University, Shanghai 200040, China; ^4^Tianqiao and Chrissy Chen Institute for Brain Diseases in Shanghai, Shanghai 200040, China

## Abstract

Claustrophobia is an anxiety disorder characterized by the fear of enclosed spaces. Although medication treatment can effectively control symptoms, the effects quickly disappear once medication is discontinued. Many studies have shown that combining psychotherapy and medication is more efficacious than solely using medication. However, the weaknesses of the traditional psychotherapy are that it is time-consuming and expensive. Alternatively, vivo exposure therapy is proposed in which anxiety is gradually triggered with stimuli. Targeting claustrophobia is diagnosed using the traditional method, and this study established virtual reality (VR) and augmented reality (AR) environments consistent with claustrophobic characteristics, comparing the two using an experimental process to examine whether VR and AR environments are equally capable of triggering anxiety in participants. This study further analysed the efficacies of VR and AR by measuring changes in participant's heart rates variability (HRV) and examining data from survey questionnaires. HRV results indicated that the proposed VR system and AR system were both able to trigger anxiety. Furthermore, the AR environment produced a stronger experience for the participants and caused physiological reactions more evident than those caused by the VR environment. Regarding the anxiety questionnaire, the participants suggested that their anxiety was significantly higher in the VR environment than in the AR environment.

## 1. Introduction

Claustrophobia is an anxiety disorder characterized by the fear of enclosed spaces [1]. Under certain circumstances, such as being in elevators, trains, or airplane cabins, sufferers can exhibit symptoms of panic or fear of panic [2]. Causes of claustrophobia are likely to be extremely small tonsils, genetic predisposition, or emotional responses induced by the classical condition. The two main symptoms are the fear of enclosed spaces and the fear of constriction [3–5]. Psychological literature suggests that people with claustrophobia do not necessarily fear the enclosed spaces themselves; instead, they fear that some dangerous event will occur in this type of environment, leading to insufficient air within the space and causing suffocation.

Cognitive therapy is a commonly accepted treatment for anxiety disorders [6]. The goal is to correct the person's misunderstanding toward the objects of their fear. A study by Rachman and Taylor [4] showed that cognitive therapy is effective in nearly 30% of individuals with claustrophobia, effectively reducing their fear and negative thoughts regarding specific environments [7, 8]. In vivo exposure therapy, which forces patients into the environments they fear, allows individuals to experience their fear. During treatment, therapists gradually increase the degree of live exposure. Booth and Rachman [7] found that live exposure therapy is effective in reducing the fear of and negative thoughts regarding enclosed spaces in nearly 75% of patients [7]. In addition to these therapies, interoceptive exposure therapy, psychoeducational intervention, reverse conditioning, and breathing retraining are somewhat effective in treating claustrophobia. In addition, antidepressants or medications used for treating high blood pressure and heart disease can reduce the discomfort felt by people with claustrophobia during anxiety attacks [7, 9–12]. Although medication treatment can effectively control symptoms, the effects quickly disappear once medication is discontinued. Many studies have shown that combining psychotherapy and medication is more efficacious than solely using medication, and that the effects of treatment are longer lasting [13]. The weaknesses of the traditional psychotherapy are that it is time-consuming [14], generally requiring 1 or 2 years to conduct a full psychiatric analysis and complete regular treatments, and expensive. Exposure therapy is similarly time-consuming, and some patients refuse returning to treatment because of the fear they experience from the method [15, 16].

Virtual reality (VR) originated from Sutherland's (1965) concept of “The Ultimate Display” [17], which uses computer simulation to produce a 3D virtual world, provides users with visual, auditory, and tactile sensory simulations and allows them to view a simulated world using computers and related equipment. VR enables real-time, unrestricted observation of objects in a 3D space and allows for user interaction. The three basic elements of VR systems for the user are immersion, interaction, and imagination [18]. It emphasizes that the user could have a better control or dominance over the virtual environment.

VR applications enjoy the advantages of simulating environments that are difficult or impossible to find in everyday life at a low cost, and these environments can be customized based on requirements to meet specific demands. It was difficult for early VR technologies to enable immersion because their graphics were unrealistic. However, years of development have led to constant innovation and improvement in both software and hardware, providing stable and reliable environments for a variety of entertainment, simulation, and training purposes. Many studies of phobias now involve experiments performed in VR [19, 20]. Instead of making a role-playing activity in real environment, VR provides physicians and patients a way for exposure therapy that is safer, more comfortable, and less resource-intensive. In addition, it is possible to use VR to construct environments that are difficult to find in real life, a feature particularly useful for patients who encounter difficulties with imaginal exposure therapy [21, 22]. Numerous research experiments have demonstrated that VR is an effective tool for treating several phobias, such as acrophobia [23], arachnophobia [24], aviophobia, claustrophobia, and agoraphobia.

Augmented reality (AR) is an approach that integrates virtual objects with the scene of real world that enables the user to perceive an augmented world, as defined by Azuma [25] and Milgram and Kishino [26]. AR technologies can calculate the spatial positions of camera images in real time and provide corresponding information through display equipment, which allows VR images to be virtually embedded in the real world and relevant interactions therein. AR applications are considerably diverse. For example, AR can provide industrial support by aiding technicians in repairing automobiles or offer more distinctive and interesting gaming experiences. In recent years, AR has been suggested to be similar to VR in its efficacy for treating phobias [27, 28]. Juan et al. [29, 30] compared AR technologies with VR for effectiveness in treating acrophobia, finding that AR and VR systems are equally capable of triggering fear of heights. In a study of VR-triggered anxiety associated with acrophobia, Juan and Pérez [31] compared the degrees of presence induced by the cave automatic virtual environment (CAVE) display and head-mounted display (HMD) devices, finding that anxiety is highly correlated with presence. Furthermore, the study showed that the degree of presence provided by the CAVE display device was significantly higher than that of the HMD.

The purpose of this study was to compare established VR and AR environments to determine whether the environments are equally capable of triggering anxiety. The triggering characteristics in VR and AR environments considered claustrophobia and some other anxiety disorder-related diseases, such as agoraphobia. Outcome measurements included heart rate variability and state/trait survey results. Yeh et al. [32] had preliminary work on the comparison of VR and AR on induced anxiety using heart rate and skin conductance as indicators of anxiety. This study presented in this paper was more advanced, while heart rate variability was measured in which more indicators in regard to anxiety were analysed. Also, some preliminary data or raw data in early stage was published [33]. This paper presented compete results with a full-scale statistical analysis.

## 2. Materials and Methods

### 2.1. System Design

This experiment compared the degree of anxiety stimulated by VR and AR environments and the degree of realism experienced by participants. An enclosed elevator was used as the simulated environment because of its relevance to everyday life and relatively high likelihood of triggering claustrophobia. In addition, an interactive virtual skyscraper elevator system was constructed using VR and AR technologies, integrating visual and auditory stimuli to trigger anxiety in the participants. The 3D virtual environment created for the proposed system was developed using the Unity 3D game engine with Windows 7 Enterprise Edition. To increase the degree of interaction between the participants and the virtual environment, the system used a Z800 3D Visor Head-Mounted Display (HMD) for the output, and an embedded posture recognition device was used in conjunction with the official software development kit (SDK) to convert users' head movements into mouse motions. Furthermore, an MSI MyECG E3-80 First Professional-Grade Portable Electrocardiogram (ECG) was employed to measure and extract the changes in the heart rates of participants during the experiment for later analysis.

**Figure 1:**
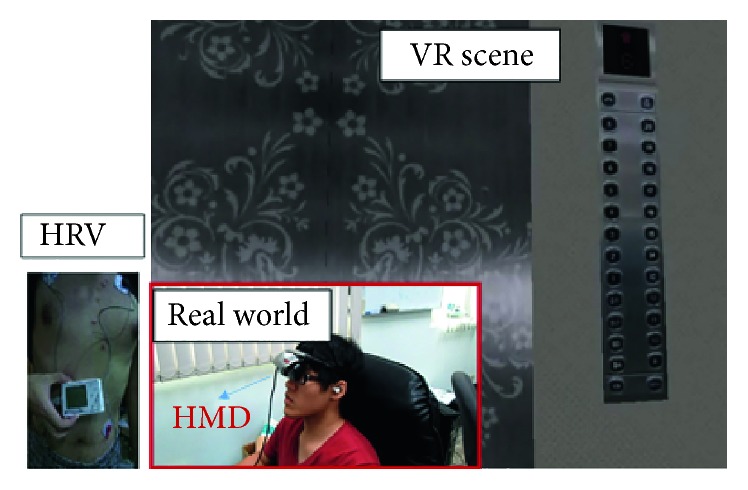
VR system.

**Figure 2:**
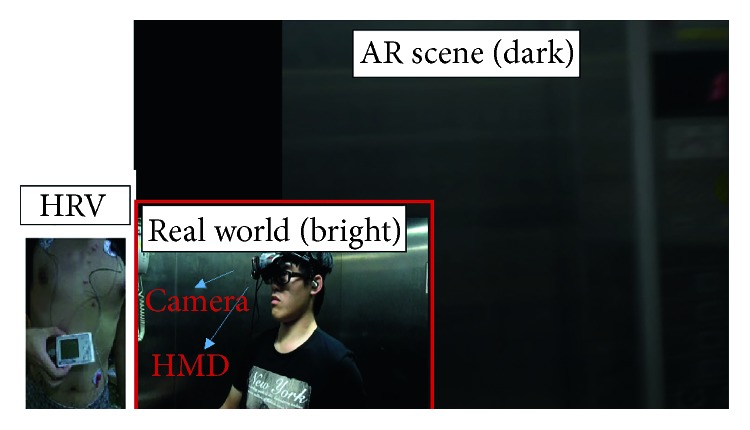
AR system.

For the VR system, all visuals were composed of virtual scenery. The 3D modules used therein were developed using 3DMAX software in conjunction with texture mapping. The modules were incorporated into Unity 3D for use after development. The system framework is shown in Figure 1. For the AR system, full high-definition images, provided by a Logitech HD Pro C910 WebCamera, were used for the visuals. The Unity 3D engine imported the live video streaming and put virtual object over the real-scene image via texture mapping in order to come out with the AR scene. Next, the researchers added stimulating incidents, such as flashing lights, an electricity blackout, a fire, and thick smoke. It was not necessary to add tags to the surrounding environment during filming; the desired effects were achieved by intuitively adding virtual objects into the environment at the appropriate locations. The system framework is shown in Figure 2.

**Figure 3:**
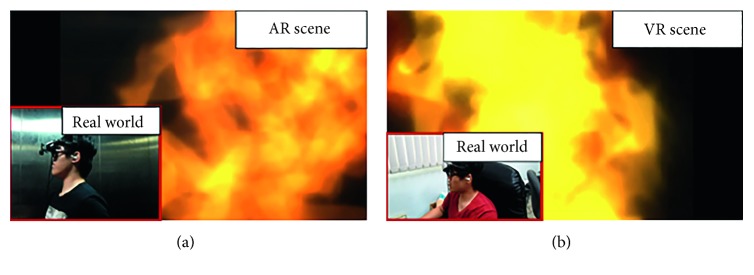
Scene with flames.

To enable adjustments in the stimulus level based on the viewer's circumstances, the following events were designed: (1) elevator door closing: when the participants enter the virtual environment, they are located in an open opaque elevator; the elevator doors suddenly close after a certain amount of time. Visually, the entire environment switches from an open to an enclosed environment; the participants are unable to see the environment outside of the elevator, generating a sense of constriction and the sound of the elevator door closing heightens the presence of the scenario. (2) Brightness level: after the elevator doors have been closed for some time, the lights inside the elevator begin to flash and then eventually turn off. Consequently, the elevator enters a state of blackout. Visually, the lights switch from light to dark, preventing the participants from seeing the objects around them and inducing a level of psychological stress. (3) Alarm sound: from an auditory perspective, fire alarms, evacuation alerts, and impact noises begin to sound, when the elevator lights begin to flash and when the elevator enters blackout, causing the participants to begin doubting the circumstances outside the elevator and eliciting fear. (4) Heartbeats sound: after the elevator enters blackout, participants cannot see any visuals for a certain period of time. A series of faint heartbeats start sounding in the darkness and, coupled with the quiet surroundings, make it difficult for participants to distinguish whether the heartbeats are sound effects or the sound of their own heartbeating. (5) Flames and heavy smoke: after participants gradually become acclimated to the surrounding darkness, flames suddenly burst out (Figure 3). Visually, the surrounding sparks and thick smoke cause the participants to believe they are in the midst of a fire. The crackling noises of the sparks add to the sense of realistic burning. (6) Screaming sounds: after the fire bursts out, participants hear screaming sounds from other virtual passengers and staff inside the building. There are four types of screams, exclamations, and cries intended to cause the participants to feel they are in an emergency situation. The frequency and type of screams alternated according to the change of the fire.

### 2.2. Experiment Design

This experiment focused on examining whether VR and AR environments elicit fear of enclosed spaces and comparing the effectiveness of these two methods as a model of claustrophobia treatment. We used heart rate variability (HRV) as an objective measure of participants' physiological status and survey questionnaires to examine the participants' experiences.

We recruited 30 participants ranging in age from 18 to 35. These participants had no medical history in regard to claustrophobia or other types of fears. The order of environment conditions was counterbalanced to reduce order effect errors. The experiment took approximately two hours to complete both conditions. The participants were first fitted with the HRV physiological data measuring the instrument to collect their normal HRV for 1 h, after which the system timestamps were synchronized for the MyECG instrument and the computer was used for the experiment. The VR/AR environmental conditions lasted 5 min each. The physiological data regarding the HRV of the participants were collected continuously. The participants were given a 10 min rest between conditions. After both conditions were completed, they were asked to complete a survey in regard with technology acceptance [34]. The SD memory cards were then removed from the MyECG instruments worn by the participants and entered into the computer for statistical analysis.

In this experiment, both the VR and AR environments were comprised of an enclosed elevator. In the VR experiment, the participants were unable to move their body after entering the environment but were allowed to rotate their heads to change their viewing angles. In the AR environment condition, to be consistent with the circumstances of the VR environment, the participants were asked to stand in the center of the elevator and to avoid making movements besides rotating their heads to view their surroundings. Baseline time was recorded as the time from the start of the experiment to the start of the first anxiety-inducing event. An equivalent baseline time was in both conditions.  Table 1 corresponds the experiment time elapsed with event occurrences.

### 2.3. Measurements

Heart rate variability (HRV) was analysed using ECGs. The analyses were divided into time domain and frequency domain. Time-domain analysis [35] uses ECG records over 24 h as the baseline data, detecting the gaps between each QRS complex wave in a continuous ECG. Adjacent R waves represent the cycles of heartbeats (i.e., the gaps or intervals between R waves (R-R)). The continuous gaps formed by consecutive R-R intervals represent HRV, defined as a normal-to-normal (NN) interval. Commonly used time-domain analyses include the following: standard deviation of the NN interval (SDNN), standard deviation of the averages of NN (SDANN), SDNN index, root mean square of successive NN interval differences (RMSSD), the percentage value of NN20 count (pNN20), and the percentage value of the NN50 count (pNN50). Because the lengths of the VR and AR environments were approximately 5 min, the researchers selected SDNN, RMSSD, pNN20, and pNN50 as the bases for comparison due to their correlation to short-term variability. The most commonly used calculation for frequency-domain analysis [35] is the fast Fourier transform (FFT), which analyzes the distribution of powers at different frequencies. Common frequency-domain analyses include total power (TP), high frequency (HF), low frequency (LF), very low frequency (VLF), ultralow frequency (ULF), normalized LF (nLF), and normalized HF (nHF). The biggest indicators of emotional influence are LF and HF. At a HF, TP of a heartbeat is subject to greater influence from the parasympathetic nervous system. Although the activity of the sympathetic nervous system increases at LF, the parasympathetic nervous system must synchronously adjust to suppress excessive excitement in the sympathetic nervous system and achieve a balanced state. Thus, LF is not necessarily directly correlated to the sympathetic nervous system. When the autonomic nervous system encounters stress from nervous emotions, activity in the sympathetic nervous system increases, whereas the opposite occurs for the parasympathetic nervous system. Therefore, this study utilized HF and log of nHF (LnHF) as the primary indicators for observation, with LnLF values serving as a supplement.

To assess whether anxiety was triggered for the participants in the VR and AR environments, a questionnaire was used to measure their degree of anxiety. This questionnaire was based on the State-Trait Anxiety Inventory and modified to utilize a 5-point scale for measuring the degree of the anxiety experienced. This scale is typically used to measure anxiety in adults. The questionnaires were divided into the VR and AR environment sections, and the participants were asked about the degree of anxiety they experienced in the VR and AR experiments. More specifically, items in questionnaires were in regard to each stimulus event (Table 1) associated with the degree of anxiety, respectively.

In addition, the technology acceptance model (TAM) [34] was used to evaluate the behavioral intentions for executing the behavior while an individual engages in a specific behavior. The TAM shows that the perceived usefulness and perceived ease of use of information technology are the two primary deciding factors for behavioral intention to use. Additionally, the perceived ease of use has a direct influence on perceived usefulness, thereby indirectly influencing intention to use. After the participants completed the task, they were asked to complete the 5-point survey items regarding their acceptance of this technology in four dimensions: awareness and presence, usefulness, ease of use, and playfulness. These dimensions represented the degree of realism of the game environment, whether the game environment was able to induce feelings of anxiety in specific scenarios, the ease of use of the game controls and the physiological feedback system, the entertainment value provided by the game, and the curiosity of the participants regarding this technology. The TAM was applied one time per participant disregarding the AR or VR system because we assumed AR and VR both laid on the same track of technology from the perspective of users.

We measured the correlation of the collected survey questionnaires using a *t*-test and performed a paired sample analysis and an independent analysis. The statistical analysis was conducted by the tool of SPSS™.

## 3. Results

### 3.1. HRV

For HRV, we divided the measured data into three main parts: data from 1 h before the experiment and data obtained during the VR and AR experiments. To compare the effectiveness of the VR and AR environments in triggering fear, we compared and analysed the normal HRV physiological data with those obtained from the VR and AR experiments. The results are shown in Tables 2 and 3. The VR and AR data were compared with the normal data, as shown in Tables 4 and 5. Finally, the VR and AR conditions' results were compared with each other, as shown in Table 6.

A comparison of the participants' HRV under normal circumstances versus HRV in the two conditions can be seen in Table 2. In the time-domain analysis, both short-term indicators (i.e., SDNN and pNN20) exhibited significant differences. In the frequency analysis, significant differences appeared in the LnHF indicator, and the other indicators approached significance. The lack of significance was likely because of an insufficient sample size; however, a descending trend was observed.

In a comparison of the means and standard deviations of the various values (Table 3), the AR conditions values were lower than normal. Because of the small sample size, only SDNN exhibited a significant decrease (Table 5). However, decreasing trends can be observed indicating that the participants were in a nervous emotional state. In the VR condition, the SDNN was again the only indicator that was significantly lower than normal (Table 4). The other indicator values were nearly equal to those in the normal state, sometimes even higher, implying that although the participants from the two conditions experienced nervousness during the VR condition, their reactions were not as pronounced as in the AR condition. As shown in Table 7, the study extracted the physiological data from the VR and AR conditions for comparison, finding that nearly all indicator values were significantly lower in the AR environment experiment than in the VR condition. This shows that anxiety was experienced more strongly by the participants in the AR condition than in the VR condition.

### 3.2. Anxiety Questionnaire

We measured the correlation of the collected survey questionnaires using a *t*-test and performed a paired sample analysis and an independent analysis (Table 8). The paired sample analysis compared the average degree of anxiety between the AR and VR environments for the 30 participants. The participants indicated that they experienced significantly greater anxiety in the VR environment than in the AR environment (Table 8).

### 3.3. TAM

In terms of the survey results for TAM (Table 6), the satisfaction was above neutral (3 points).

## 4. Discussion

For the HRV, as shown in the data in Tables 4 [Table tab5] [Table tab6] [Table tab7]–8, the AR condition generated better results than the VR condition. This finding may be due to that the participants in the AR condition were physically present in an actual environment, causing them to experience a more natural presence and become more engaged. The effects of the VR condition were inconspicuous, possibly because the participants did not suffer from claustrophobia and therefore showed less-pronounced reactions. Although the HMDs achieved immersive surround effects, previous studies [6, 7] have shown that HMDs are limited in their ability to achieve surround effects compared to other surround displays, such as CAVE displays, resulting in a poorer sense of presence and posing challenges for participants in immersing themselves in the virtual environment.

Regarding the anxiety questionnaire, the participants suggested that their anxiety was significantly higher in the VR condition compared to the AR condition. This finding substantially differed from the HRV physiological data measured. Practically speaking, however, these results are not improbable. Anxiety involves both psychological and physiological factors. Although participants may not have felt psychologically nervous, they may have experienced physiological reactions in response to the stimuli. Physiological signals are more objective data and were synchronously measured during the experiment. By contrast, the questionnaire responses were subjective, naturally creating a possible discrepancy. Nevertheless, the two sets of results are not necessarily contradictory. Because the participants recruited for this study did not suffer from claustrophobia, the simple act of entering an elevator scenario was not likely to cause substantial subjective emotional fluctuation. Thus, the participants may have overlooked their own anxious emotions.

Although the experiment utilized sufficient display equipment and used HMDs to create surround visuals and immersive effects, the overall average score of presence was only 3.50. This result was similar to that achieved by Juan and Pérez in a study comparing the presence and degree of anxiety induced by HMD and CAVE [29] devices. Their study showed that the presence of HMD was a mean of 3.59 (out of a maximum of seven points), slightly higher than that of normal circumstances. Usefulness was the second-highest scoring item in the TAM survey (mean = 3.85). The participants exhibited a positive attitude toward the use of the HMD, suggesting that the HMD helped them perceive their correct positions in the virtual environment, increasing the quality and effects of the VR task. In contrast, ease of use was the lowest-scoring item (mean 3.38). The researchers inferred that the HMD and HRV instruments were relatively unfamiliar to the participants, requiring instructions regarding use. After instructions were provided, the participants quickly learned how to use the equipment, requiring only one round of operation. Finally, the average overall score for playfulness was 3.86, the highest scoring item for TAM in this experiment.

In this experiment, the researchers identified a number of challenges regarding the VR and AR conditions. Regarding the display equipment, the VR conditions required better audiovisual effects to create an immersive experience (as with the CAVE display device). In contrast, the AR conditions required integration with their surroundings, requiring consideration of equipment for sound and light effects, as well as an emphasis on maneuverability. In addition, regarding AR conditions, participants must be physically present in the environment, leading to differences in presence when compared to VR conditions. Although the two conditions utilized the same equipment and stimuli, other factors may influence the experience of users, such as changes in temperature, standing posture, and external noises beyond the control of the researchers. Assessing the presence of AR environments may require a different approach than that for VR environments to allow participants to adequately evaluate their experiences.

In summary, the researchers found that, in this experiment, the AR environment produced a statistically significantly stronger experience for the participants and caused statistically significant physiological reactions than those caused by the VR environment. However, in clinical therapy for claustrophobia, AR environment experiments are more difficult to construct than are VR environments. Furthermore, patients have a lower degree of acceptance during exposure therapy. Therefore, in conjunction with developing AR-based therapy, improving VR display equipment to provide greater presence may help induce the anxiety associated with enclosed spaces and therefore an opportunity to provide an intervention for claustrophobia.

## 5. Conclusions

This study successfully developed virtual reality (VR) and augmented reality (AR) environments for claustrophobia treatments. A test was conducted to validate these two systems and examine the effect between these two systems using HRV and anxiety questionnaires. HRV results indicated that the proposed VR system and AR system were both able to trigger anxiety. Furthermore, the AR environment produced a stronger experience for the participants and caused statistically significant physiological reactions than those caused by the VR environment. Regarding the anxiety questionnaire, the participants suggested that their anxiety was significantly higher in the VR environment than those in the AR environment. In the future, a large-scale clinical test is planned to further verify the therapeutic effect of the proposed systems.

## Figures and Tables

**Table 1:** Script of events.

Time elapsed for the game		Event occurred
Time 0	Baseline	Start of first stimulus
Time 1	After 30 s	Elevator door opens
Time 2	After 45 s	Elevator door closes
Time 3	After 55 s	Lights flash and alarms activate within the elevator
Time 4	After 1 min 40 s	Lights turn out, and evacuation sounds can be heard
Time 5	After 2 min 5 s	Collision sound and heartbeating sound can be heard
Time 6	After 3 min 40 s	Flames rise, and screaming sounds can be heard
Time 7	5 min	Simulation ends

**Table 2:** One-way ANOVA of the HRV physiological data values.

		Sum of squares	Degree of freedom	Mean sum of squares	*F*	Significance
SDNN	Between-group	9123.245	2	4561.622	12.253	0.000
	Within-group	32389.664	87	372.295		
	Overall	41512.909	89			

RMSSD	Between-group	1298.241	2	649.120	2.559	0.083
	Within-group	22067.628	87	253.651		
	Overall	23365.869	89			

pNN20	Between-group	2518.745	2	1259.372	6.138	0.003
	Within-group	17851.371	87	205.188		
	Overall	20370.116	89			

pNN50	Between-group	897.829	2	448.914	2.563	0.083
	Within-group	15236.540	87	175.133		
	Overall	16134.369	89			

HF	Between-group	11054376.62	2	5527188.311	2.601	0.080
	Within-group	184856864.5	87	2124791.546		
	Overall	195911241.1	89			

LnHF	Between-group	3.891	2	1.945	3.478	0.035
	Within-group	48.662	87	0.559		
	Overall	52.553	89			

LnLF	Between-group	1.244	2	0.622	2.457	0.092
	Within-group	22.016	87	0.253		
	Overall	23.259	89			

**Table 3:** Descriptive statistics for the HRV physiological data between groups.

		Quantity	Mean	SD
SDNN	Normal	30	80.81	24.90
	AR	30	56.19	12.23
	VR	30	67.21	18.64

RMSSD	Normal	30	48.19	14.29
	AR	30	42.82	12.99
	VR	30	52.08	19.70

pNN20	Normal	30	47.90	12.95
	AR	30	42.67	15.30
	VR	30	55.55	14.62

pNN50	Normal	30	21.51	11.53
	AR	30	18.45	12.01
	VR	30	26.13	15.76

HF	Normal	30	2025.77	1330.49
	AR	30	1536.63	1285.64
	VR	30	2392.17	1717.94

LnHF	Normal	30	7.40	0.70
	AR	30	7.04	0.79
	VR	30	7.53	0.75

LnLF	Normal	30	7.77	0.44
	AR	30	7.49	0.47
	VR	30	7.65	0.59

**Table 4:** Comparison of HRV physiological data between VR and normal circumstances.

	Quantity	*t*-value	Significance (two-tailed)
SDNN	30	2.396	0.020
RMSSD	30	−0.875	0.385
pNN20	30	−2.144	0.036
pNN50	30	−1.298	0.199
HF	30	−0.924	0.360
LnHF	30	−0.706	0.483
LnLF	30	0.935	0.353

**Table 5 tab5:** Comparison of HRV physiological data between AR and normal circumstances.

	Quantity	*t*-value	Significance (two-tailed)
SDNN	30	4.861	0.000
RMSSD	30	1.525	0.133
pNN20	30	1.431	0.158
pNN50	30	1.006	0.319
HF	30	1.448	0.153
LnHF	30	1.866	0.067
LnLF	30	2.452	0.017

**Table 6 tab6:** TAM results.

	Awareness + presence	Usefulness	Ease of use	Playfulness
Mean score	3.50	3.86	3.38	3.56

**Table 7 tab7:** Comparison of HRV physiological data between VR and AR.

	Quantity	*t*-value	Significance (two-tailed)
SDNN	30	−2.706	0.009
RMSSD	30	−2.150	0.036
pNN20	30	−3.335	0.001
pNN50	30	−2.124	0.038
HF	30	−2.184	0.033
LnHF	30	−2.467	0.017
LnLF	30	−1.176	0.244

**Table 8:** Paired sample analysis for anxiety.

Group	Sample size	Mean	SD	*t*-value	Significance
AR mean	30	3.16	0.55	−4.29	^*∗∗*^
VR mean	30	3.58	0.48		

Significance level = 0.05; ^*∗∗*^indicates *P* < 0.01.

## Data Availability

The heart rate variability data used to support the findings of this study are available from the corresponding author upon request.
